# Blockade of TIPE2-Mediated Ferroptosis of Myeloid-Derived Suppressor Cells Achieves the Full Potential of Combinatory Ferroptosis and Anti-PD-L1 Cancer Immunotherapy

**DOI:** 10.3390/cells14020108

**Published:** 2025-01-13

**Authors:** Hafiza Kashaf Tariq, Zihao Liang, Lawan Rabiu, Abdulrahman Ibrahim, Nada Mohamady Farouk Abdalsalam, Rong Li, Qiong Yang, Xiaochun Wan, Dehong Yan

**Affiliations:** 1 Guangdong Immune Cell Therapy Engineering and Technology Research Center, Center for Protein and Cell-Based Drugs, Institute of Biomedicine and Biotechnology, Shenzhen Institutes of Advanced Technology, Chinese Academy of Sciences, Shenzhen 518055, China; hafizakashaftariq@siat.ac.cn (H.K.T.); zh.liang@siat.ac.cn (Z.L.); rabiu@siat.ac.cn (L.R.); abdulrahman@siat.ac.cn (A.I.); nadamohamady@siat.ac.cn (N.M.F.A.); r.li5@siat.ac.cn (R.L.); 2 University of Chinese Academy of Sciences, Beijing 100864, China; 3 School of Medicine, South China University of Technology, Guangzhou 510006, China; yangq@scut.edu.cn

**Keywords:** TIPE2, MDSCs, ferroptosis, immune checkpoint blockade, cancer treatment

## Abstract

Although immune checkpoint blockade (ICB) therapy has attained unprecedented clinical success, the tolerance and immune suppression mechanisms evolved by tumor cells and their tumor microenvironment (TME) hinder its maximum anti-cancer potential. Ferroptosis therapy can partially improve the efficacy of ICB, but it is still subject to immune suppression by myeloid-derived suppressor cells (MDSCs) in the TME. Recent research suggests that an MDSC blockade can unleash the full therapeutic potential of the combined therapy of ferroptosis and ICB in liver cancer treatment. However, whether blocking the intrinsic ferroptosis pathways of MDSCs can relieve imidazole ketone erastin (IKE)-initiated ferroptosis-induced immune suppression and ultimately trigger the optimal therapeutic effect of the combined ferroptosis and ICB therapy is still unknown. Here, we report that TIPE2, a phospholipid transfer protein, regulated the ferroptosis susceptibility in MDSCs through reprogramming lipid peroxidation-related phosphatidylethanolamine (PE) and phosphatidylcholine (PC) species composition. TIPE2-deficient MDSCs resisted IKE-induced ferroptosis by up-regulating SLC7A11 and GPX4, and dissolved ferroptosis-induced immunosuppressive function by down-regulating lipid ROS whilst encouraging T cell proliferation and infiltration into tumor tissues to improve ferroptosis therapy. More importantly, TIPE2-deficient MDSCs achieved the full anti-tumor therapeutic potential of IKE-induced ferroptosis therapy and a PD-L1 blockade. These findings indicate that TIPE2 confers the ferroptosis sensitivity of MDSCs, and combining the targeting of the TIPE2 of MDSCs, ferroptosis therapy, and ICB is a novel therapeutic option for cancer treatment.

## 1. Introduction

Immune checkpoint blockade (ICB) therapy has had notable clinical success over the past ten years since the FDA approved the first immune checkpoint inhibitor in 2011, and has revolutionized the way cancer treatment is conducted [1,2]. Although the unprecedented long-lasting clinical responses have been achieved with ICBs such as anti-PD-L1 antibody immunotherapy, only a small subset of patients with specific tumor types like melanoma, lung cancer, and colorectal cancer can respond to and obtain therapeutic benefits [1,2,3,4]. In addition, a number of patients initially respond to ICB, but eventually develop resistance to this therapy [2,3,4]. The advantage of ICB therapy is that it reactivates and restores the killing activity of cytotoxic T cells (CTLs) against tumor cells. Nevertheless, it underestimates the tolerance mechanisms evolved by tumor cells based on the progression of treatment, or the inhibitory effect of the tumor microenvironment on T cell therapy, ultimately leading to resistance to ICB treatment.

To fulfill the needs of rapid growth and proliferation, tumor cells undergo metabolic reprogramming to utilize and release lipids in TME so as to adapt to the low oxygen microenvironment [5,6]. Such a lipid-rich and hypoxic microenvironment, on the one hand, can inhibit the anti-tumor T cell response, but on the other hand, it can also expose the weakness of tumor cells to ferroptosis [7,8,9,10]. Ferroptosis is a novel form of regulated cell death distinct from apoptosis and pyroptosis, which is driven by iron, reactive oxygen species (ROS), and lipid peroxidation in cells [9,10,11,12,13]. In fact, lipid peroxidation is a biochemistry process in which ROS oxidizes the highly active double bonds of arachidonic acid (C20:4), adrenic acid (C22:4), docosahexaenoic acid (22:6) or linoleic acid (C18:2) [10,13,14,15]. Acyl-coenzyme A synthetase long-chain family member 4 (ACSL4) can mediate the synthesis of polyunsaturated fatty acid (PUFA)-containing phospholipid (PUFA-PL) through catalyzing the incorporation of PUFAs into membrane phospholipids [13,14,16,17,18]. Thus, PUFA-PLs such as phosphatidylethanolamine (PE) and phosphatidylcholine (PC) species containing C18:0/C20:4, C18:0/C22:4, and C18:0/C22:6 are susceptible to ROS-induced peroxidation, which leads to the excessive accumulation of toxic lipid peroxides on cellular membranes, disrupting cellular permeability and membrane function [13,14,16]. To mitigate these toxic effects of lipid peroxides, cells have evolved their defense systems to detoxify lipid peroxides, mainly including the solute carrier family 7-member 11 (SLC7A11)-glutathione peroxidase 4 (GPX4) system [19,20,21,22,23,24,25]. Extracellular cystine is imported into cells by SLC7A11 and then cystine is converted to cysteine in the cytosol. Cysteine is then converted to reduced glutathione (GSH), which is utilized by GPX4 as the cofactor to reduce lipid peroxides to non-toxic lipid alcohols. If the buffering capacity of these cellular defenses is exceeded, the accumulation and degradation of PUFA-PL peroxides induces ferroptosis.

In addition to the central SLC7A11-GPX4 ferroptosis defense systems, other ferroptotic defense frameworks mainly include the ferroptosis suppressor protein 1 (FSP1), the dihydroorotate dehydrogenase (DHODH), the GTP cyclohydrolase 1 (GCH1), and the solute carrier family 40 member 1 (SLC40A1) system [5,26,27,28,29]. Interestingly, except ACSL4, there are also other ferroptosis-driving mechanisms, for instance, voltage-dependent anion-selective channel protein 2 (VDAC2), phosphorylase kinase G2 (PHKG2), nicotinamide adenine dinucleotide phosphate (NADPH) oxidase 1 (NOX1), spermidine/spermine N^1^-acetyltransferase 1 (SAT1), cation transport regulator homolog 1 (CHAC1), cysteinyl-tRNA synthetase (CARS), heme oxygenase 1 (HO-1) [13,23,30,31,32,33,34]. The pharmacological inhibition or genetic inactivation of ferroptosis-driving genes like ACSL4 can resist ferroptosis, but the genetic loss or chemical inhibition of ferroptosis defense systems, such as blocking SLC7A11 through imidazole ketone erastin (IKE) or prohibiting GPX4 by RAS synthetic lethal 3 (RSL3), can favor ferroptosis [17,20,22,24,25]. Actually, RSL3 induces lipid peroxidation and ferroptosis in a panel of tumor cell lines in vitro, and IKE also increases the lipid peroxidation of tumor cells and inhibits tumor growth in different immunodeficient murine models [11,12,20,21,22,24,35]. Moreover, CD8^+^ T cells can trigger ACSL4-dependent tumor ferroptosis by utilizing IFN-γ and arachidonic acid, thus elevating ICB cancer immunotherapy [17,36,37]. Further, the uptake of arachidonic acid and oxidized lipids by the scavenger receptor CD36 in tumor-infiltrating CD8^+^ T cells can induce lipid peroxidation and ferroptosis, leading to reduced cytotoxic cytokine production and impairing anti-tumor function [7,8]. More incredibly, the ferroptosis inducers, RSL3 and IKE, significant attract CD8^+^ T cell ferroptosis but inhibit CD8^+^ T cell proliferation and function, thus increasing tumor progression in immunocompetent mice [7,8,38,39]. These studies demonstrate that current therapeutic strategies targeting the ferroptosis of tumor cells may damage tumor-infiltrating CD8^+^ T cells in an off-target way, thus leading to the failure of immunotherapy.

Besides tumor metabolites, immune suppressive cells in TME are also key factors leading to poor ICB therapy, in which myeloid-derived suppressive cells (MDSCs) are the main limiting factor [40,41]. MDSCs (CD45^+^CD11b^+^ Gr-1^+^) are a diverse population of immature myeloid cells which can be separated into two main groups in murine tumor tissues: monocytic MDSCs (M-MDSCs, CD45^+^CD11b^+^Ly6G^−^Ly6C^+^) and polymorphonuclear MDSCs (PMN-MDSCs, CD45^+^CD11b^+^Ly6G^+^Ly6C^−^). MDSCs can provoke T cell dysfunction by depleting the extracellular accessibility of L-arginine via the arginase 1 (ARG1)-dependent metabolic pathway, and dysregulate T cell receptor (TCR) signaling via ROS-evoked oxidative stress [6,40,42]. Through these inhibitory activities, MDSCs exert potent immunosuppressive effects on CTLs and shield tumor cells from immune killing, thus encouraging a pro-tumor environment. Indeed, lipid metabolism is a primary source of energy and confers the immunosuppressive function of MDSCs in TME [43]. Tumor MDSCs increase the uptake of exogenous PUFAs like linoleic acid to enhance ROS release, which intensifies their ability to inhibit T cell immunity, thereby accelerating the growth of tumors [44]. Lipid buildup in tumors causes MDSCs to go through metabolic reprogramming via glycolysis to fatty acid oxidation (FAO) and oxidative phosphorylation, to fulfill the survival demand [43,44]. Likewise, this preference for lipid metabolism makes MDSCs prone to ferroptosis [45,46]. The inducible ferroptosis of MDSCs through activating the p53-Hmox1 signaling pathway inhibits MDSC aggregation, but increases T cell infiltration and thus enhances CD8^+^ T cell-mediated tumor suppression in immunocompetent tumor-bearing mice [47]. Yet, it is worth noting that the IKE induction of ferroptosis of MDSCs via absorbing oxidized PL-PUFAs by arachidonate 12/15-lipoxygenase (Alox12/15), ACSL4, or fatty acid transport protein 2 (FATP2) triggers lipid peroxidation and the release of a large number of PGE2, contributing to immunosuppressive activity against antitumor T cells in immunocompetent murine tumor models [6,38,45,46,48,49]. These seemingly contradictory results suggest that multiple intrinsic pathways can regulate MDSC ferroptosis, and different pathways could differentially impact context-dependent T cell response and the final efficacy of tumor immunotherapy. Interestingly, a current study suggests that the triple combination of the ferroptosis-inducing agent, MDSC blockade, and ICB therapy can unleash the maximum therapeutic potential in liver cancer [50]. However, the way in which the MDSC blockade mediates the molecular mechanism of tumor suppression is not yet known.

TIPE2, a member of tumor necrosis factor-α–induced protein 8-like (TNFAIP8L) family, is a phospholipid transfer protein that can bind phosphoinositide species such as phosphatidylinositol 4,5-bisphosphate (PIP2) and phosphatidylinositol 3,4,5- trisphosphate (PIP3) and shuttle phospholipids to and from the plasma membrane in macrophages and neutrophils [51,52]. TIPE2 upregulation in tumor cells can cause cell death and significantly reduce Ras-induced tumorigenesis in mouse models [52]. TIPE2 sustains the inhibitory function of MDSCs targeting T cells in tumor-bearing mice, as TIPE2 serves as a crucial molecular switch controlling MDSC functional polarization through conversion between M2 immunosuppressive signature genes like ARG-1 and M1 proinflammatory signatures, such as inducible nitric oxide synthase (iNOS) [53]. In addition, tumor-derived ROS induce TIPE2 expression in MDSCs, but TIPE2-deficient MDSCs lowers ROS release, suggesting that TIPE2 can govern ROS production through a positive feedback loop in MDSCs [53]. However, whether TIPE2 contributes to the ferroptosis induction of MDSCs by shaping phospholipid composition and triggering lipid peroxidation remains to be characterized. Here, we provide evidence that TIPE2 confers the ferroptosis sensitivity of MDSCs and TIPE2-deficient MDSCs achieve the full potential of ferroptosis therapy and PD-L1 blockade.

## 2. Materials and Methods

### 2.1. Experimental Mice

C57BL/6 mice (6–8 weeks old) were purchased from Zhuhai Bestest Biotechnology Co., Ltd. (Zhuhai, China). These animals were housed under specific-pathogen-free (SPF) conditions in the Animal Facilities of the Shenzhen Institute of Advanced Technology (SIAT), Chinese Academy of Sciences (CAS), with a 12 h light–dark cycle.TIPE2-deficient (TIPE2^−/−^) mice on a C57BL/6J background were generated as previously described [54].

### 2.2. Cell Lines and Culture

Murine tumor cell lines, including LLC (Lewis lung carcinoma), B16F10 (melanoma), and MC38 (colorectal carcinoma), were obtained from the CAS cell bank. Cells were cultured in DMEM supplemented with 10% FBS and 1% penicillin–streptomycin (PS) and maintained at 37 °C in a humidified incubator with 5% CO₂. Medium changes were performed every 2–3 days, and cells were passaged at approximately 90% confluence.

### 2.3. Reagents and Antibodies

RPMI-1640 medium, DMEM, PBS, FBS, and PS were procured from VivaCell (Shanghai, China). IKE was sourced from TargetMol (Boston, MA, USA). Zombie NIR Fixable Viability Kit, CFSE, and PI were purchased from BioLegend (San Diego, CA, USA) and Invitrogen (Carlsbad, CA, USA). Antibodies used for flow cytometry included CD45, CD11b, Gr-1, F4/80, CD11c, CD3, CD4, CD8, CD19, NK1.1, CD25, and CD127 (all from BioLegend, San Diego, CA, USA). Western blotting antibodies were specific to ARG-1 (R&D Systems, Minneapolis, MN, USA), iNOS (CST, Danvers, MA, USA), GPX4 and SLC7A11 (Abcam, Cambridge, MA, USA), TIPE2, ACSL4, ACTIN (Proteintech, Wuhan, China), and VINCULIN (Santa Cruz Biotechnology, Santa Cruz, CA, USA).

### 2.4. MDSCs Isolation and Sorting

Bone marrow cells were isolated from the femurs and tibias of WT and TIPE2^−/−^ mice, and red blood cells were lysed using ACK buffer. For the in vitro generation of MDSCs, 2 × 10⁶ bone marrow cells were cultured in RPMI-1640 containing 10% FBS, 1% PS, 40 ng/mL GM-CSF, and 40 ng/mL IL-6. Fresh cytokine-containing medium was added on the 3rd day, and cells were collected on 4th day. MDSCs were enriched using Biotin anti-mouse Gr-1 antibody and Streptavidin Nanobeads (BioLegend, San Diego, CA, USA), with purity > 90% confirmed by flow cytometry.

### 2.5. Lipidomic Analysis of MDSCs

1 × 10⁷ Gr-1⁺ MDSCs were isolated from tumor tissues of LLC TB WT and TIPE2^−/−^ mice treated with IKE (5 mice per group), washed with PBS, and lipids were extracted following the Bligh and Dyer method. Lipid extracts were dissolved in 90% isopropanol/acetonitrile and subjected to high-performance liquid chromatography (HPLC, Shimadzu LC-30AD)-MS/MS (AB SCIEX QTRAP 6500+, SCIEX)). Lipids were separated on a HILIC column (Kinetex C18 2.6 um, 100 A°, 100 mm × 2.1 mm; Phenomenex, Torrance, CA, USA ) with a mobile phase gradient of acetonitrile–water and ammonium acetate. Data were analyzed using SCIEX OX software 3.0 (SCIEX, Framingham, MA, USA), with relative lipid abundances normalized to internal standards (EquiSplash; Avanti Polar Lipids, Alabaster, AL, USA).

### 2.6. Tumor Leukocyte Isolation and Flow Cytometry

Tumors harvested from LLC-bearing WT and TIPE2^−/−^ mice were dissected under sterile conditions and digested in a mixture of Collagenase I, II, IV, and hyaluronidase (Sigma, St. Louis, MO, USA) at 37 °C for 1 h. Single-cell suspensions were stained with Zombie NIR viability dye and fluorochrome-conjugated antibodies. Data were acquired using a Cytek^®^ Aurora flow cytometer and analyzed with SpectroFlo software (https://cytekbio.com/pages/spectro-flo).

### 2.7. Cell Viability Assay

Gr-1^+^ MDSCs extracted from the bone marrow or tumor were incubated with different concentrations of ferrostatin-1 (1 µM), necrostatin-1 (1 µM), VAD-FMK (zVAD, 10 µM), and RSL3 (1 µM, 5 µM, 10 µM, and 20 µM) for 24 h. AlamarBlue Cell Viability Reagent (Invitrogen) was used to assess cell viability.

### 2.8. The Ferroptosis Markers Detection

The expression of lipid level, lipid uptake, lipid peroxidation, reactive oxygen species (ROS), and nitric oxide (NO) were measured by PI staining with 500 ug/mL BODIPY 493/503 (Invitrogen, Carlsbad, CA, USA), 10 ug/mL BODIPY FLC16 (Invitrogen, Carlsbad, CA, USA), 500 ug/mL BODIPY 581/591 C11 (Invitrogen, Carlsbad, CA, USA), 5 μM chloromethyl-2, 7-dichlorofluorescein diacetate (CM-H_2_DCFDA, Invitrogen, Carlsbad, CA, USA), and 5 μM Diaminofluorescein-FM diacetate (DAF-FM DA, Beyotime, Shanghai, China), respectively.

### 2.9. qRT-PCR

Total RNA was extracted using RNAiso Plus reagent and converted into cDNA using a Takara kit (Takara bio. Inc.,Tokyo, Japan). Real-time PCR was performed using SYBR Green on a BIO-RAD (Hercules, CA, USA) CFX96 system. Gene expression was normalized to β-actin, and relative levels were calculated using the 2^−ΔΔCt^ method. The qRT-PCR was performed as previously described [55]. All primers were obtained from Genewiz (Suzhou, China) and are displayed in Table S1.

### 2.10. Western Blotting

Using RIPA lysis buffer, the tumor Gr1^+^ MDSCs was lysed on ice. To find the protein concentration, cell lysates were centrifuged at 12,000× *g* for 10 min at 4 °C. The supernatant was then collected. For electrophoresis, one sample of SDS buffer was added to the supernatant. Protein blotting was performed using 30 µg of protein per lane and a pre-stained molecular weight protein marker separated on a 10% or 12% SDS-PAGE gel made using an SDS-PAGE kit (Genefist, Shanghai, China). The protein was then electro-transferred to an immunoblot PVDF membrane (Beyotime, Shanghai, China). The membrane was blocked for at least an hour in a fast Western block kit (Beyotime, Shanghai, China), and then it was gently agitated and incubated with primary antibodies for the entire night at 4 °C. The housekeeping proteins that were used were vinculin and β-actin. The membrane was incubated for an hour with HRP-conjugated secondary antibodies, and then the protein was detected using enhanced chemiluminescence (ECL) Western blotting substrate and observed on an Amersham Imager 600 (GE Healthcare, Little Chalfont, Buckinghamshire, UK).

### 2.11. T Cell Proliferation Assay

Naïve CD3⁺ T cells were isolated from splenocytes of WT C57BL/6 mice using CD3-negative selection kits (BioLegend, San Diego, CA, USA). Purified T cells were stained with CFSE and activated with anti-CD3/CD28-coated beads (BioLegend, San Diego, CA, USA). Gr-1^+^ MDSCs were extracted from the tumors of LLC TB mice treated with drugs (40 µM IKE and 1mM L-NAC), washed extensively with complete media after 6 h, and then co-cultured with CFSE-labeled CD3^+^ T cells at the ratio of 1:1, 1:2, and 1:4 for 72 h at 37 °C. CFSE dilution was analyzed using flow cytometry, with decreased fluorescence intensity indicating T cell proliferation.

### 2.12. Establishment of In Vivo Tumor Models

C57BL/6 WT or TIPE2^−/−^ mice were injected subcutaneously with 2 × 10⁶ LLC, B16F10, or MC38 cells in the lower right flank. Tumor volume was measured every other day using a digital caliper and calculated as 0.5 × (length) × (width^2^). Mice were treated with IKE (10 mg/kg) or a combination of IKE (10 mg/kg) and anti-PD-L1 (200 μg) from day 7 post-tumor implantation for two weeks. Tissues were harvested for further analysis.

### 2.13. Statistical Analysis

Data are presented as mean ± SD. Statistical significance was evaluated using GraphPad Prism 8.4.2 software (Graphpad Software, La Jolla, CA, USA). Comparisons were made using one-way or two-way ANOVA or unpaired Student’s t-tests, with significance denoted as *p* ≤ 0.05, *p* ≤ 0.01, or * *p* ≤ 0.001.

## 3. Results

### 3.1. TIPE2 Regulated Ferroptosis Susceptibility of MDSCs

**Figure 1:**
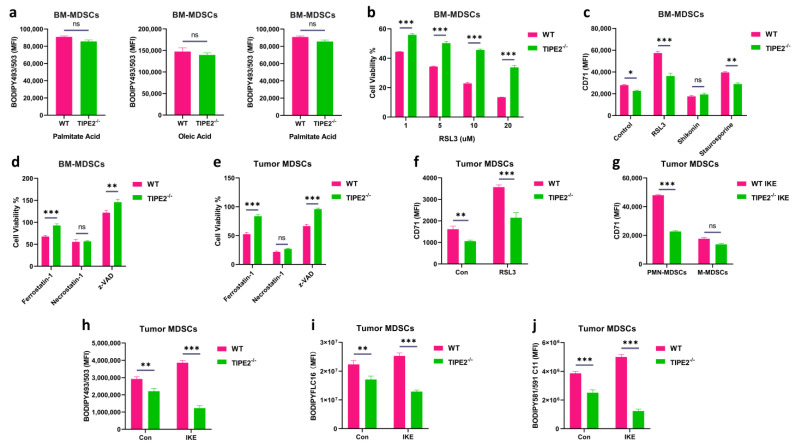
TIPE2 is involved in the induction of ferroptosis in MDSCs. (**a**) Fatty acid accumulation with lipophilic fluorescent dye BODIPY493/503 in Gr−1^+^ MDSCs extracted from the bone marrow treated with palmitate acid (saturated fatty acid, 100 μM), linoleic acid (polyunsaturated fatty acid, 100 μM), and oleic acid (monounsaturated fatty acid, 100 μM) for 48 h. Mean ± SD are shown. (**b**) AlamarBlue cell viability assay on Gr−1^+^ MDSCs extracted from the bone marrow incubated with different concentrations 1 µM, 5 µM, 10 µM, and 20 µM of RSL3 for 24 h. Mean ± SD are shown. (**c**) CD71 expression in Gr−1^+^ MDSCs extracted from the bone marrow in the presence of different cell death inducers for 18 h: ferroptosis (RSL3, 20 µM), necroptosis (Shikonin, 1 µM), and apoptosis (Staurosporine, 0.25 µM). Mean ± SD are shown. (**d**) AlamarBlue cell viability assay on Gr-1^+^ MDSCs extracted from the bone marrow in the presence of different cell death inhibitors for 24 h: 1 µM ferrostatin-1 for ferroptosis, 1 µM necrostatin−1 for necroptosis, and 10 µM z−VAD−FMK (zVAD) for apoptosis. Mean ± SD are shown. (**e**) AlamarBlue cell viability assay on Gr−1^+^ MDSCs extracted from the LLC TB mice in the presence of different cell death inhibitors for 24 h: 1 µM ferrostatin-1 for ferroptosis, 1 µM necrostatin-1 for necroptosis, and 10 µM z−VAD−FMK (zVAD) for apoptosis. Mean ± SD are shown. (**f**) CD71 expression in Gr−1^+^ MDSCs extracted from the LLC TB mice in the presence of ferroptosis inducer 10 µM RSL3 for 24 h. Mean ± SD are shown. (**g**) CD71 expression in PMN−MDSCs and M−MDSCs extracted from the LLC TB mice. Mean ± SD are shown. (**h**) Lipid level with lipophilic fluorescent dye BODIPY493/503 in Gr−1^+^ MDSCs extracted from the LLC TB mice treated with IKE. Mean ± SD are shown. (**i**) Lipid uptake with palmitic acid fluorescent dye BODIPYFLC16 in Gr−1^+^ MDSCs extracted from the LLC TB mice treated with IKE. Mean ± SD are shown. (**j**) Lipid peroxidation level with fluorescent dye BODIPY 581/591 C11 in Gr−1^+^ MDSCs extracted from the LLC TB mice treated with IKE. Mean ± SD are shown. (**a**–**j**) Data are expressed as *, *p* ≤ 0.05, **, *p* ≤ 0.01, ***, *p* ≤ 0.001, or ns, no significant difference.

TIPE2, a phospholipid transfer protein, can influence cellular lipid composition by moving the phospholipid between the cell membrane and cytoplasm [52]. Lipid peroxidation is typically characterized by the accumulation of PE and PC species containing C18:0/C20:4, C18:0/C22:4, and C18:0/C22:6 in cells [14]. To test whether TIPE2 changes lipid peroxidation-related PE and PC species in MDSCs, we measured these PUFA-PLs and PUFA-ePLs in tumor MDSCs of Wild-type (WT) and TIPE2-deficient (TIPE2^−/−^) C57BL/6 mice bearing LLC (Lewis lung carcinoma) treated with IKE (the ferroptosis inducer widely used for in vivo research) by targeted phospholipid analysis using the HPLC-MS/MS system. This analysis showed that the content of either PE (plasm-18:0/20:4), PE (plasm-18:0/22:4), or PE (18:0/22:6), but not PE (18:0/20:4) and PE (18:0/22:4), was lower in TIPE2^−/−^ tumor MDSCs than in WT tumor MDSCs (where plasm indicates plasmalogens or PUFA-ePLs). Among the choline-containing phospholipids, the content of either PC (18:0/20:4), PC (18:0/22:4), or PC (18:0/22:6) was also reduced in TIPE2^−/−^ tumor MDSCs than in WT tumor MDSCs (Table 1). PUFA-PLs and PUFA-ePLs are formed through the uptake and accumulation of exogenous fatty acids in cells. To identify whether TIPE2 regulates fatty acid accumulation in MDSCs, we detected the lipid level in Gr-1^+^ bone marrow-derived MDSCs (BM-MDSCs) treated with different fatty acids in the in vitro culture. We found that TIPE2^−/−^ MDSCs markedly inhibited polyunsaturated fatty acid (linoleic acid) accumulation but did not affect the level of saturated fatty acids (palmitate acid) and monounsaturated fatty acids (oleic acid) compared to WT MDSCs (Figure 1a and Figure S1a).

To elucidate whether TIPE2 regulates ferroptosis sensitivity in the in vitro MDSCs, we first ensured that RSL3 (ferroptosis inducer) induced the cell death of WT BM-MDSCs in a dose-dependent manner, but TIPE2 deficiency protected BM-MDSCs from RSL3-triggered cell death (Figure 1b). The transferrin receptor CD71 is a marker of ferroptosis [46]. As expected, RSL3, neither Shikonin (necroptosis inducer) nor Staurosporine (apoptosis inducer) significantly upregulated CD71 expression in WT BM-MDSCs, but TIPE2^−/−^ BM-MDSCs decreased RSL3-induced CD71 expression compared to WT BM-MDSCs (Figure 1c and Figure S1b). Similarly to previous reports that apoptosis and ferroptosis contribute to the death of MDSCs, the viability of TIPE2^−/−^ BM-MDSCs was improved by zVAD (apoptosis inhibitor) or by ferrostatin-1 (ferroptosis inhibitor), but not by necrostatin-1 (necroptosis inhibitor) when compared to WT BM-MDSCs treated with the indicated inhibitors (Figure 1d). Likewise, either zVAD or ferrostatin-1, but not necrostatin-1, increased the viability of TIPE2^−/−^ tumor MDSCs compared with WT tumor MDSCs (Figure 1e). Similarly, RSL3 markedly augmented CD71 level in WT tumor MDSCs, but TIPE2 deletion blocked the RSL3-upregulated CD71 expression of tumor MDSCs (Figure 1f).

We further investigated whether TIPE2 regulates ferroptosis susceptibility in the in vivo MDSCs; WT and TIPE2^−/−^ LLC tumor-bearing (TB) mice were treated with IKE to induce ferroptosis in TME, and then the MDSCs extracted from tumor tissues were detected by flow cytometry. We observed that TIPE2 loss notably reduced the CD71 expression in IKE-treated tumor PMN-MDSCs compared to IKE-treated WT tumor PMN-MDSCs, but TIPE2 deficiency did not change the CD71 expression of IKE-treated tumor M-MDSCs compared to IKE-treated WT tumor M-MDSCs (Figure 1g and Figure S1c). TIPE2 deletion greatly decreased the lipid accumulation (Figure 1h) and uptake (Figure 1i) level of IKE-treated tumor MDSCs when compared to IKE-treated WT tumor M-MDSCs. More importantly, when we measured the lipid peroxidation by using BODIPY 581/591 C11 dye, we found that a TIPE2 defect tremendously inhibited the lipid peroxidation in IKE-treated tumor MDSCs compared with IKE-treated WT tumor M-MDSCs (Figure 1j and Figure S1d). Collectively, these data demonstrate that TIPE2 regulated ferroptosis susceptibility in the in vitro and in vivo MDSCs through reprogramming lipid peroxidation-related PE and PC species composition.

### 3.2. TIPE2 Specified Ferroptosis Gene Expression Pattern of MDSCs

**Figure 2:**
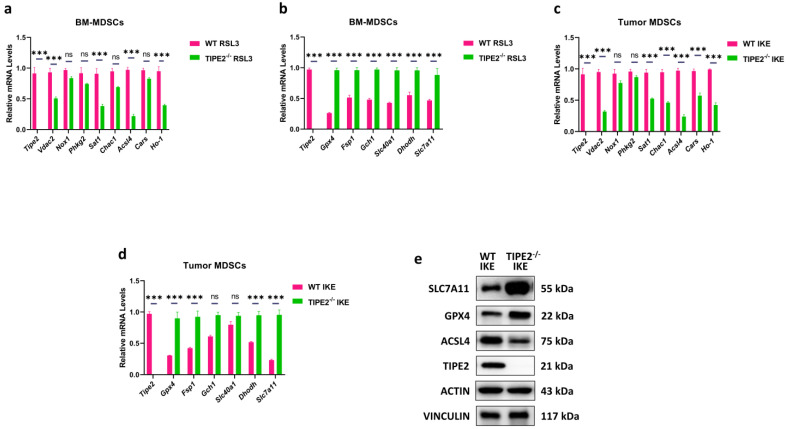
TIPE2 regulates ferroptosis-related genes of MDSCs. (**a**) mRNA expression of ferroptosis-promoting genes in MDSCs. qRT−PCR was conducted in Gr−1^+^ MDSCs extracted from the bone marrow and treated with RSL3. Mean ± SD are shown. (**b**) mRNA expression of ferroptosis resistance genes in MDSCs. qRT−PCR was conducted in Gr−1^+^ MDSCs extracted from the bone marrow and treated with RSL3. Mean ± SD are shown. (**c**) mRNA expression of ferroptosis−promoting genes in MDSCs. qRT-PCR was conducted in Gr−1^+^ MDSCs extracted from the LLC TB mice treated with IKE. Mean ± SD are shown. (**d**) mRNA expression of ferroptosis resistance genes in MDSCs. qRT−PCR was conducted in Gr−1^+^ MDSCs extracted from the LLC TB mice treated with IKE. Mean ± SD are shown. (**e**) Protein expression of ferroptosis−promoting and resistance genes in MDSCs. Western blot was conducted in Gr−1^+^ MDSCs extracted from the LLC TB mice treated with IKE. Mean ± SD are shown. (**a**–**d**) Data are expressed as ***, *p* ≤ 0.001, or ns, no significant difference.

To elucidate the underlying mechanism by which TIPE2 affects the ferroptosis sensitivity of MDSCs, we used RT-qPCR method to screen the key genes of ferroptosis-driving and ferroptosis defense systems of the in vitro and in vivo MDSCs. Our results showed that RSL3-treated TIPE2^−/−^ BM-MDSCs revealed a significant decrease in the ferroptosis-driving genes such as *Vdac2*, *Sat1*, *Acsl4* and *Ho-1*, but did not affect the other ferroptosis-driving genes like *Nox1*, *Phkg2*, *Chac1* and *Cars* compared to RSL3-treated WT BM-MDSCs (Figure 2a). On the contrary, RSL3-treated TIPE2^−/−^ BM-MDSCs displayed a markedly higher level of ferroptosis defense genes such as *Gpx4*, *Fsp1*, *Gch1*, *Slc40a1*, *Dhodh*, and *Slc7a11* than RSL3-treated WT BM-MDSCs (Figure 2b). Similarly, IKE-treated TIPE2^−/−^ tumor MDSCs also significantly decreased the expression of *Vdac2*, *Sat1*, *Acsl4*, *Ho-1*, *Chac1*, and *Cars* genes, but showed no difference in the expression of *Nox1* and *Phkg2* genes compared to IKE-treated WT tumor MDSCs (Figure 2c). Instead, IKE-treated TIPE2^−/−^ tumor MDSCs expressed remarkably higher levels of *Gpx4*, *Fsp*1, *Dhodh*, and *Slc7a11* genes than IKE-treated WT tumor MDSCs, but no statistical differences in the expression of *Gch1* and *Slc40a1* genes were noted between IKE-treated TIPE2^−/−^ tumor MDSCs and IKE-treated WT tumor MDSCs (Figure 2d). We further confirmed by western blotting that IKE-treated TIPE2^−/−^ tumor MDSCs profoundly enhanced the key ferroptosis defense proteins SLC7A11 and GPX4, but immensely lowered the main lipid peroxidation trigger protein ACSL4 when compared to IKE-treated WT tumor MDSCs (Figure 2e and Figure S2). Collectively, these findings show that the lack of TIPE2 converted MDSCs from a susceptible phenotype to a resistance phenotype to ferroptosis via specifying the ferroptosis gene expression pattern.

### 3.3. TIPE2 Conferred Ferroptosis-Induced Immunosuppressive Function in MDSCs

**Figure 3:**
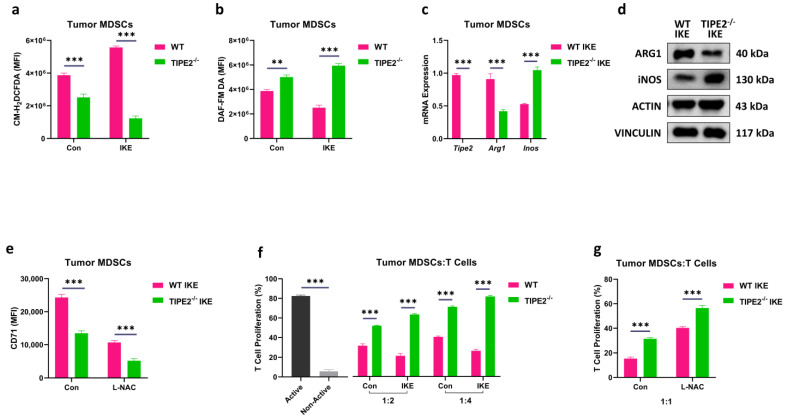
TIPE2−deficient MDSCs decrease ferroptosis−induced suppressive function. (**a**) ROS level using the CM−H_2_DCFDA fluorescent dye in Gr−1^+^ extracted from the LLC TB mice treated with IKE. Mean ± SD are shown. (**b**) NO level using the DAF−FM DA fluorescent dye in Gr−1^+^ MDSCs extracted from the LLC TB mice treated with IKE. Mean ± SD are shown. (**c**) mRNA expression of *Arg1* and *Inos* in MDSCs. qRT−PCR conducted in Gr−1^+^ MDSCs extracted from the LLC TB mice treated with IKE. Mean ± SD are shown. (**d**) Protein expression of ARG1 and iNOS in MDSCs. Western blot conducted in Gr−1^+^ MDSCs extracted from the LLC TB mice treated with IKE. Mean ± SD are shown. (**e**) CD71 expression in Gr-1^+^ extracted from the LLC TB mice treated with ROS inhibitor (L−NAC, 1 mM). Mean ± SD are shown. (**f**) The expression of T cell proliferation in Gr−1^+^ MDSCs extracted from the LLC TB mice treated with 40 µM IKE (ferroptosis inducer) for 6 h, washed extensively, and cocultured with CFSE−labeled CD3^+^ T cells at the ratio of 1:2 and 1:4. Mean ± SD are shown. (**g**) The expression of T cell proliferation in Gr−1^+^ MDSCs extracted from the LLC TB mice treated with ROS inhibitor (L−NAC, 1 mM) and cocultured with CFSE−labeled CD3^+^ T cells at the ratio of 1:1. Mean ± SD are shown. (**a**–**c**,**e**–**g**) Data are expressed as **, *p* ≤ 0.01, or ***, *p* ≤ 0.001.

Previous work has indicated that ferroptosis renders MDSCs more immunosuppressive [46]. To investigate whether TIPE2 confers ferroptosis-induced immunosuppressive function in MDSCs, we checked the key immunosuppressive mediators of MDSCs. Due to decreased lipid peroxidation-related phospholipid accumulation, IKE-treated TIPE2^−/−^ tumor MDSCs unquestionably reduced the ROS production when compared to IKE-treated WT tumor MDSCs (Figure 3a and Figure S3a). Surprisingly, IKE-treated TIPE2^−/−^ tumor MDSCs substantially increased the nitric oxide (NO) level compared to IKE-treated WT tumor MDSCs (Figure 3b and Figure S3a). Through RT-qPCR and western blotting detection, we observed that IKE-treated TIPE2^−/−^ tumor MDSCs evidently decreased the M2 immunosuppressive signature gene (*Arg1*) and marker protein (ARG1), whereas they markedly increased the M1 immune stimulatory signature gene (*Inos*) and protein (iNOS) when compared to IKE-treated WT tumor MDSCs (Figure 3c,d and Figure S3d). Furthermore, treatment with the ROS inhibitor N-acetyl cysteine (L-NAC) led to a more significant reduction in CD71 expression in IKE-treated TIPE2^−/−^ tumor MDSCs compared to IKE-treated WT tumor MDSCs (Figure 3e and Figure S3b). Indeed, when tested in vitro, IKE-treated TIPE2^−/−^ tumor MDSCs had a reduced capacity to block T cell proliferation in a ratio-dependent manner, compared with those from IKE-treated WT controls (Figure 3f and Figure S3c). Importantly, IKE-treated TIPE2^−/−^ tumor MDSCs pretreated with L-NAC further reduced their ability to block T cell proliferation, compared with those from IKE-treated WT controls (Figure 3g and Figure S3c). The above results suggest that TIPE2 conferred the ferroptosis-induced immunosuppressive function of MDSCs in a lipid ROS-dependent manner.

### 3.4. TIPE2-Deficient MDSCs Sensitized Tumor to Ferroptosis Therapy via Turning Cold into Hot Tumor

**Figure 4:**
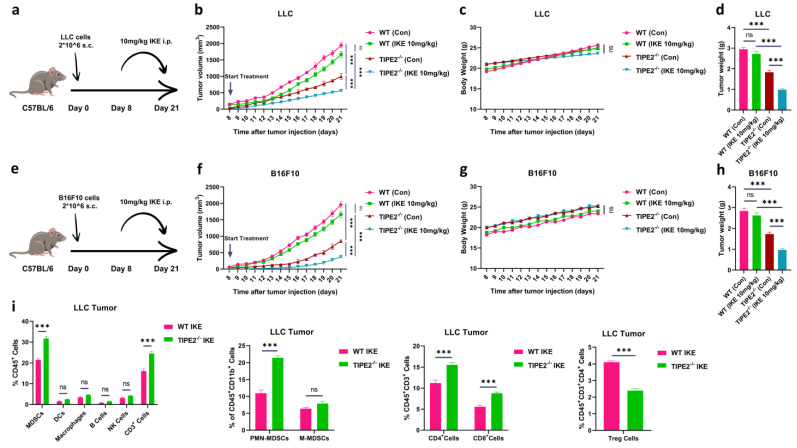
TIPE2-deficient MDSCs enhance ferroptosis-induced tumor growth inhibition via reprogramming the immune microenvironment. (**a**) A schematic representation of the experimental design; C57BL/6 mice were injected s.c. with LLC cells on day 0. Then, IKE (10mg/kg) was injected i.p. at day 8 till day 21. (**b**) The tumor volume of LLC C57BL/6 mice (n = 3 mice/group) treated with IKE for 2 weeks. Mean ± SD are shown. (**c**) The tumor weight of LLC C57BL/6 mice (n = 3 mice/group) treated with IKE for 2 weeks. Mean ± SD are shown. (**d**) The body weight of LLC C57BL/6 mice (n = 3 mice/group) treated with IKE for 2 weeks. Mean ± SD are shown. (**e**) A schematic representation of the experimental design, C57BL/6 mice were injected s.c. with B16F10 cells on day 0. Then, IKE (10 mg/kg) was injected i.p. at day 8 till day 21. (**f**) The tumor volume of B16F10 C57BL/6 mice (n = 3 mice/group) treated with IKE for 2 weeks. Mean ± SD are shown. (**g**) The tumor weight of B16F10 C57BL/6 mice (n = 3 mice/group) treated with IKE for 2 weeks. Mean ± SD are shown. (**h**) The body weight of B16F10 C57BL/6 mice (n = 3 mice/group) treated with IKE for 2 weeks. Mean ± SD are shown. (**i**) The percentages of immune cells in tumor tissues extracted from the LLC TB mice treated with IKE: MDSCs, PMN-MDSCs, M-MDSCs, Treg cells, DCs, macrophages, B cells, NK cells, CD3^+^ T cells, CD4^+^ T cells, CD8^+^ T cells, were discovered by flow cytometry. MDSCs were CD45^+^ CD11b^+^ Gr-1^+^ cells; PMN-MDSCs were CD45^+^ CD11b^+^ Ly6C^−^Ly6G^+^ cells; M-MDSCs were CD45^+^ CD11b^+^ Ly6C^+^ Ly6G^−^ cells; Treg cells were CD45^+^ CD3^+^ CD4^+^ CD25^+^ CD127^−^ cells; DCs were CD45^+^ CD11b^+^ CD11c^+^ cells; macrophages were CD45^+^CD11b^+^ F4/80^+^ cells; B cells were CD45^+^CD3^−^ CD19^+^ cells; and NK cells were CD45^+^ CD3^−^ NK1.1^+^ cells; CD3^+^ T cells were CD45^+^ CD3^+^ cells; CD4^+^ T cells were CD45^+^ CD3^+^CD4^+^ CD8^−^ cells; CD8^+^ T cells were CD45^+^CD3^+^CD4^−^CD8^+^ cells. Mean ± SD are shown. (**b**–**d**,**f**–**i**) Data are expressed as ***, *p* ≤ 0.001, or ns, no significant difference.

Previous research has indicated that the induction of ferroptosis by IKE treatment does not limit the tumor growth in immunocompetent CT26 (colon carcinoma)-bearing mice [46]. In addition, TIPE2 deficiency disabled the ferroptosis-induced immunosuppressive function of MDSCs (Figure 3f,g). We wanted to explore whether TIPE2-deficient MDSCs sensitized tumors to ferroptosis therapy. We compared the tumor growth kinetics of WT and TIPE2^−/−^ C57BL/6 mice subcutaneous (s.c.)-injected LLC (Lewis lung carcinoma) or B16F10 (melanoma) cells with or without IKE treatment. IKE was administered daily, while the drugs were given starting from day 8 post-tumor inoculation into the LLC or B16F10 tumor-bearing mice (Figure 4a,e). Consistent with previous reports, IKE treatment alone failed to inhibit the tumor growth in both tumor models and TIPE2 deletion alone delayed the tumor progression. However, IKE treatment further significantly retarded tumor growth in TIPE2^−/−^ LLC or B16F10 tumor-bearing mice (Figure 4b,f). The tumor growth inhibition did not alter the body weight in TIPE2^−/−^ LLC or B16F10 tumor-bearing mice compared with the other group (Figure 4c,g). In addition, the maximum reduction in tumor weight and size was observed in IKE-treated TIPE2^−/−^ LLC or B16F10 tumor-bearing mice compared with the other group (Figure 4d,h and Figure S4e,f). Similarly, in MC38 (colorectal carcinoma) tumor-bearing mice (Figure S4a), the smallest tumor volume, weight, and size were noted in IKE-treated TIPE2^−/−^ mice compared with the other group (Figure S4b,d,g), and no significant difference in the body weight of all the mice was observed (Figure S4c).

To investigate the immunological mechanism underlying the anti-tumor therapeutic effect of ferroptosis treatment sensitized by TIPE2-deficient MDSCs, we checked the distribution of immune cells in the tumor tissues of WT and TIPE2^−/−^ LLC tumor-bearing mice with IKE treatment. Using flow cytometric techniques, we found that the percentages of several CD45^+^ subsets, including MDSCs, PMN-MDSCs, CD3^+^ T cells, CD4^+^ T cells, and CD8^+^ T cells, were prominently increased, but the percentages of Tregs were significantly decreased in IKE-treated tumors of TIPE2^−/−^ LLC-bearing mice compared with those of WT controls (Figure 4i and Figure S5a–c). However, the percentages of M-MDSCs, dendritic cells (DCs), macrophages, B cells, and natural-killer (NK) cells in IKE-treated tumors were not different between LLC-bearing WT and TIPE2^−/−^ mice (Figure 4i and Figure S5a–c). Our previous research has shown that TIPE2 deletion can polarize MDSCs from an immunosuppressive state into a stimulatory one. In IKE-induced ferroptosis context, we also confirmed that TIPE2^−/−^ MDSCs already transformed into immunostimulatory myeloid cells (Figure 3). Thus, in IKE-treated TIPE2^−/−^ LLC-bearing mice, increasing the accumulation of immunostimulatory MDSCs can promote effector CD4^+^ and CD8^+^ T cells to infiltrate into the tumor tissues, but restrain the immunosuppressive Tregs. As a conclusion, TIPE2-deficient MDSCs sensitized tumor to ferroptosis therapy by turning cold tumors into hot tumors.

### 3.5. TIPE2-Deficient MDSCs Achieved the Full Potential of Ferroptosis Therapy and PD-L1 Blockade

**Figure 5:**
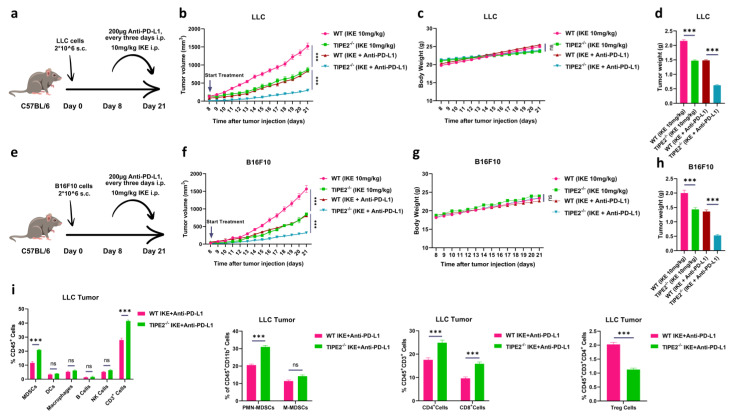
The ferroptosis therapy of TIPE2−deficient MDSCs enhances anti−PD−L1 cancer immunotherapy. (**a**) A schematic representation of the experimental design C57BL/6 mice were injected s.c. with LLC cells on day 0. Then, IKE (10mg/kg) was injected i.p. at day 8 till day 21. Also, anti−PD−L1 (200 μg) was injected i.p. every 3 days. (**b**) The tumor volume of LLC C57BL/6 mice (n = 3 mice/group) treated with IKE for 2 weeks. Mean ± SD are shown. (**c**) The tumor weight of LLC C57BL/6 mice (n = 3 mice/group) treated with IKE for 2 weeks. Mean ± SD are shown. (**d**) The body weight of LLC C57BL/6 mice (n = 3 mice/group) treated with IKE for 2 weeks. Mean ± SD are shown. (**e**) A schematic representation of the experimental design C57BL/6 mice were injected s.c. with B16F10 cells on day 0. Then, IKE (10mg/kg) was injected i.p. at day 8 till day 21. Also, anti−PD−L1 (200 μg) was injected i.p. every 3 days. (**f**) The tumor volume of B16F10 C57BL/6 mice (n = 3 mice/group) treated with IKE for 2 weeks. Mean ± SD are shown. (**g**) The tumor weight of B16F10 C57BL/6 mice (n = 3 mice/group) treated with IKE for 2 weeks. Mean ± SD are shown. (**h**) The body weight of B16F10 C57BL/6 mice (n = 3 mice/group) treated with IKE for 2 weeks. Mean ± SD are shown. (**i**) The percentages of immune cells in tumor tissues extracted from the LLC TB mice treated with IKE: MDSCs, PMN−MDSCs, M−MDSCs, Treg cells, DCs, macrophages, B cells, NK cells, CD3^+^ T cells, CD4^+^ T cells, CD8^+^ T cells, were discovered by flow cytometry. MDSCs were CD45^+^ CD11b^+^ Gr−1^+^ cells; PMN−MDSCs were CD45^+^ CD11b^+^ Ly6C^−^Ly6G^+^ cells; M−MDSCs were CD45^+^ CD11b^+^ Ly6C^+^ Ly6G^−^ cells; Treg cells were CD45^+^ CD3^+^ CD4^+^ CD25^+^ CD127^−^ cells; DCs were CD45^+^ CD11b^+^ CD11c^+^ cells; macrophages were CD45^+^CD11b^+^ F4/80^+^ cells; B cells were CD45^+^CD3^−^ CD19^+^ cells; and NK cells were CD45^+^ CD3^−^ NK1.1^+^ cells; CD3^+^ T cells were CD45^+^ CD3^+^ cells; CD4^+^ T cells were CD45^+^ CD3^+^CD4^+^ CD8^−^ cells; CD8^+^ T cells were CD45^+^CD3^+^CD4^−^CD8^+^ cells. Mean ± SD are shown. (**b**–**d**,**f**–**i**) Data are expressed as ***, *p* ≤ 0.001, or ns, no significant difference.

Considering that ICB therapy mainly contributes to restoring the killing function of CTLs against tumor cells in tumor tissues [1], and TIPE2 deletion in MDSCs can re-sensitize tumors to ferroptosis therapy (Figure 4), we wanted to examine whether TIPE2-deficient MDSCs combined with ferroptosis induction and ICB therapy could represent a therapeutic option for tumor treatment. WT and TIPE2^−/−^ mice bearing s.c. LLC or B16F10 tumors were administrated with IKE (one injection every day), or anti-PD-L1 antibody (one injection every three days), or a combination of IKE and anti-PD-L1 antibody for two weeks starting from day 8 post-tumor inoculation (Figure 5a,e). While the dual administration of IKE and anti-PD-L1 antibody inhibited LLC or B16F10 tumor growth compared to a single administration of IKE in WT mice, the combination of these two showed even more effectiveness in delaying LLC or B16F10 tumor progression in TIPE2^−/−^ mice when compared with the dual treatment in WT mice (Figure 5b,f). The combination therapeutic effect was irrelevant to body weight (Figure 5c,g), but was directly correlated with tumor weight and mass (Figure 5d,h and Figure S5d,e); that is to say, the better the combination therapeutic effect, the lower tumor weight and mass.

To investigate the immunological mechanism underlying the anti-tumor therapeutic effect of combined IKE and anti-PD-L1 antibody treatment enhanced by TIPE2-deficient MDSCs, we checked the distribution of immune cells in the tumor tissues of WT and TIPE2^−/−^ LLC tumor-bearing mice with dual administration. By flow cytometric analysis, we found that the percentages of MDSCs, PMN-MDSCs, CD3^+^ T cells, CD4^+^ T cells and CD8^+^ T cells were evidently raised, but the percentages of Tregs were noticeably reduced in double combination-treated tumors of TIPE2^−/−^ LLC-bearing mice compared with those of WT controls (Figure 5i). However, the percentages of M-MDSCs, DCs, macrophages, B cells, and NK cells in dual combination-treated tumors were comparable between LLC-bearing WT and TIPE2^−/−^ mice (Figure 5i). Collectively, these data suggest that TIPE2-deficient MDSCs could achieve the full potential of ferroptosis therapy and PD-L1 blockade.

## 4. Discussion

Here, we report that TIPE2 mediates the IKE-induced ferroptosis of MDSCs through the accumulation of lipid peroxidation-related phospholipids. However, TIPE2-deficient MDSCs resist IKE-induced ferroptosis through up-regulating the SLC7A11-GPX4 ferroptosis defense systems, and relieve ferroptosis-induced immunosuppressive function by down-regulating ARG1 while promoting T cell proliferation and infiltration into tumor tissues to enhance ferroptosis therapy. More importantly, TIPE2-deficient MDSCs achieve the full anti-tumor therapeutic potential of IKE-induced ferroptosis therapy and the PD-L1 blockade.

TIPE2 is the lynchpin that confers the ferroptosis sensitivity of MDSCs through metabolic reprogramming lipid peroxidation-related phospholipids. Actually, TIPE2 protein has a hydrophobic pocket with a TIPE2-homology (TH) domain which binds to an array of phospholipids, such as phosphatidylinositol 3,5-bisphosphate, phosphatidylinositol 3,4-bisphosphate, phosphatidylinositol 3-phosphate, phosphatidylinositol 5-phosphate, phosphatidic acid, PIP2, PIP3, and PE. TIPE2 shuttles PIP2 and PIP3 to the plasma membrane to potentiate phosphoinositide-3 kinase (PI3K) signaling, and then recruits AKT for signal transduction [52]. Previous studies have shown that the activation of PI3K-phospho-AKT (S473)-mechanistic target of rapamycin (mTOR)C1 signaling induces sterol regulatory element-binding protein 1 (SREBP1)-stearoyl-CoA desaturase-1 (SCD1)-mediated monounsaturated fatty acid (MUFA)-containing phospholipid (MUFA-PL) production in the plasma membrane, thus leading to the suppression of ferroptosis in tumor cells [56]. Significantly, TIPE2 deletion selectively increases phospho-AKT (S473) in MDSCs [53]. Additionally, in this study, TIPE2 deficiency reduced PUFA uptake as well as the accumulation of lipid peroxidation-related PE and PC species, including PE (plasm-18:0/20:4), PE (plasm-18:0/22:4), PE (18:0/22:6), PC (18:0/20:4), PC (18:0/22:4) or PC (18:0/22:6), the ACSL4 protein level, and subsequent lipid ROS production in tumor MDSCs. These results suggest that TIPE2 may activate PI3K-phospho-AKT (T308, not S473)-ACSL4 signaling pathway for PUFA-PL production. These excessive accumulations of toxic lipid peroxides on MDSC membranes in turn shattered the SLC7A11-GPX4 ferroptosis defense systems, thus rendering MDSCs sensitive to ferroptosis. Yet, the loss of TIPE2 may activate the PI3K-phospho-AKT(S473, not T308)-SCD1 signaling pathway for MUFA-PL production. This could destroy the balance between MUFA-PLs and PUFA-PLs, which may determine the susceptibility of MDSCs to ferroptosis. However, the possible molecular mechanisms by which TIPE2 regulates phospholipid composition in MDSCs need to be further investigated.

Our results indicate that TIPE2 determines the ferroptosis-induced immunosuppressive function in MDSCs. Our previous research has shown that exogenous ROS in TME could stimulate the TIPE2 expression of MDSCs by cytomembrane-anchored p47^phox^, a key component of NADPH oxidase 2 (NOX2), and this in turn activates the key CCAAT/enhancer-binding protein-β (C/EBPβ) signaling pathway to release immunosuppressive cytokine IL-6 in MDSCs, thereby inhibiting T cell responses in tumor [53]. In this study, we proved that TIPE2 conferred ferroptosis-induced immunosuppressive function of MDSCs in lipid ROS-dependent way, considering that other lipid transport proteins Alox12/15, ACSL4, or FATP2 also regulate MDSC ferroptosis sensitivity by shaping cellular phospholipid composition and further promote MDSC immunosuppressive activity by lipid ROS along with PGE2 [6,46,49,57,58]. These results suggest that multiple intrinsic lipid metabolism pathways control the ferroptosis sensitivity and ferroptosis-induced immunosuppressive function of MDSCs and TIPE2 is a novel endogenous ferroptosis regulator in MDSCs. Targeting TIPE2 in MDSCs not only induces MDSC sensitivity to ferroptosis, but also removes the immunosuppressive TME, thereby further enhancing cancer ferroptotic therapy.

Additionally, our study suggests that the triple combination of targeting the TIPE2 of MDSCs, ferroptosis induction, and ICB therapy is a novel therapeutic option for cancer treatment. In our study, TIPE2 deficiency disabled the ferroptosis-induced immunosuppressive function of MDSCs. In IKE-treated TIPE2^−/−^ LLC tumor-bearing mice, increasing the accumulation of immunostimulatory MDSCs can promote effector CD4^+^ and CD8^+^ T cells, not immunosuppressive Tregs, to infiltrate into the tumor tissues. Thus, TIPE2-deficient MDSCs sensitized tumors to ferroptosis therapy via turning cold tumors into hot tumors. In such a hot tumor environment, ICB therapy can restore the maximum anti-cancer potential of CTLs, thereby unleashing the full therapeutic potential of this triple combination therapy. This finding is consistent with earlier reports that targeting MDSC infiltration achieves the full potential of ferroptosis induction and PD-1 blockade [50]. However, there are still many challenges in specifically targeting TIPE2 in MDSCs in a cancer treatment clinical setting. Firstly, TIPE2 is a cytoplasmic protein that can shuttle between the intracytoplasmic membrane and cytoplasm [52]. This makes it difficult for large molecule drugs such as antibodies to target the intracellular molecule TIPE2. Recently, UM-164, a small molecule drug that can enter cells, was found to be a potential TIPE2 inhibitor [59]. However, the targeted inhibition of TIPE2 in MDSCs using UM-164 has not yet been confirmed in preclinical and clinical settings. Secondly, TIPE2 is expressed in myeloid and lymphoid immune cells and non-immune cells like hepatocytes, neurons, and epithelial cells [52]. This may lead to the non-targeted side effects of systemic administration of interfering RNA drugs such as TIPE2 siRNA in clinical practice, although this technique has been widely used in preclinical research. Notably, nanomaterials offer promising solutions to facilitate drug delivery targeting the intracellular molecule of MDSCs [60]. Nevertheless, there are currently no reports on the application of nanomaterials for delivering TIPE2 inhibitors or siRNA. Finally, TIPE2 could act both as a tumor suppressor and tumor activator [61,62]. On the one hand, the overexpression of TIPE2 in tumor cells induced cell death, proving that it might be a tumor suppressor. However, we analyzed the data for TIPE2 and CD8 mRNA expression in human lung adenocarcinoma in the TCGA database using the cBioPortal website tools. The results showed that there was a direct positive relationship between TIPE2 and CD8 mRNA expression in human lung adenocarcinoma in the TCGA database, but the altered TIPE2 expression tumor group had lower overall survival probability than unaltered tumor group (Figure S6). On another hand, TIPE2 expression in MDSCs contributed to tumor immunosuppression, thus promoting tumor progression. However, the data were not available to determine the relationship between the expression of TIPE2 and CD8 in the MDSCs of the same lung adenocarcinoma set in the TCGA database. Although the TCGA tumor data shows positive relationship between TIPE2 and CD8, our preclinical data indicate that TIPE2 loss in MDSCs dictates the response to the combination therapy of the ferroptosis activator and immune therapy (Figure 5). Also, lung cancer patients with TIPE2 high-level expression in MDSCs had poorer long-term survival than the ones with TIPE2 low-level expression in MDSCs [53]. How to solve the issue, which selectively targeting TIPE2 in MDSCs did not affect its expression in tumor cells, deserves more consideration, and needs more clinical trials to verify it.

## 5. Conclusions

In conclusion, these results provide new perspectives for the advancement of a novel therapeutic strategy aimed at disrupting TIPE2-induced ferroptosis resistance in MDSCs to render tumors sensitive to ferroptosis and ICB therapy, with promising clinical implications. Targeting TIPE2 highlights a previously overlooked strategy synergistical with ferroptosis induction and PD-L1 blockade in melanoma and lung carcinoma.

## Figures and Tables

**Table 1:** Targeted lipid peroxidation-related phospholipid analysis.

Lipid Name	Sample Content (ng/10^7^cells)	
	Wild-Type Tumor MDSCs	TIPE2^−/−^ Tumor MDSCs
PE(plasm-18:0/20:4)	168.202	150.256
PE(plasm-18:0/22:4)	189.163	161.832
PE(18:0/20:4)	261.241	273.473
PE(18:0/22:4)	98.037	104.530
PE(18:0/22:6)	24.261	21.589
PC(18:0/20:4)	269.301	237.701
PC(18:0/22:4)	72.865	59.113
PC(18:0/22:6)	36.357	30.562

## Data Availability

The original contributions presented in the study are included in the article. Further inquiries can be directed to the corresponding authors.

## Supplementary Materials

The following supporting information can be downloaded at: https://www.mdpi.com/article/10.3390/cells14020108/s1. Figure S1. TIPE2 contributes to the MDSCs’ activation of ferroptosis. Figure S2. Immunoblot membrane findings demonstrating TIPE2 regulation of ferroptosis-related genes of MDSCs. Figure S3. TIPE2-deficient MDSCs reduced ferroptosis-induced suppressive effect. Figure S4. TIPE2-deficient MDSCs enhance ferroptosis-induced tumor growth inhibition. Figure S5. TIPE2-deficient MDSCs enhance ferroptosis-induced tumor growth inhibition with anti-PD-L1 cancer immunotherapy via reprogramming immune microenvironment. Figure S6. There was a direct positive relationship between TIPE2 and CD8 mRNA expression in human lung adenocarcinoma in the TCGA database, but the tumor group with altered TIPE2 expression had lower overall survival probability than the unaltered tumor group. Table S1: Primer sequences used for qPCR analyzing the gene expressions in in vitro and in vivo Gr1^+^ MDSCs.
