# Supplementary material for: Blockade of TIPE2-Mediated Ferroptosis of Myeloid-Derived Suppressor Cells Achieves the Full Potential of Combinatory Ferroptosis and Anti-PD-L1 Cancer Immunotherapy
Source: Cells. 2025 Jan 13;14(2):108. doi: 10.3390/cells14020108 (PMC11763990; doi:10.3390/cells14020108)
Supplement: Supplementary file 1 [file cells-14-00108-s001.zip › cells-3372561-supplementary.pdf]

Supplementary data

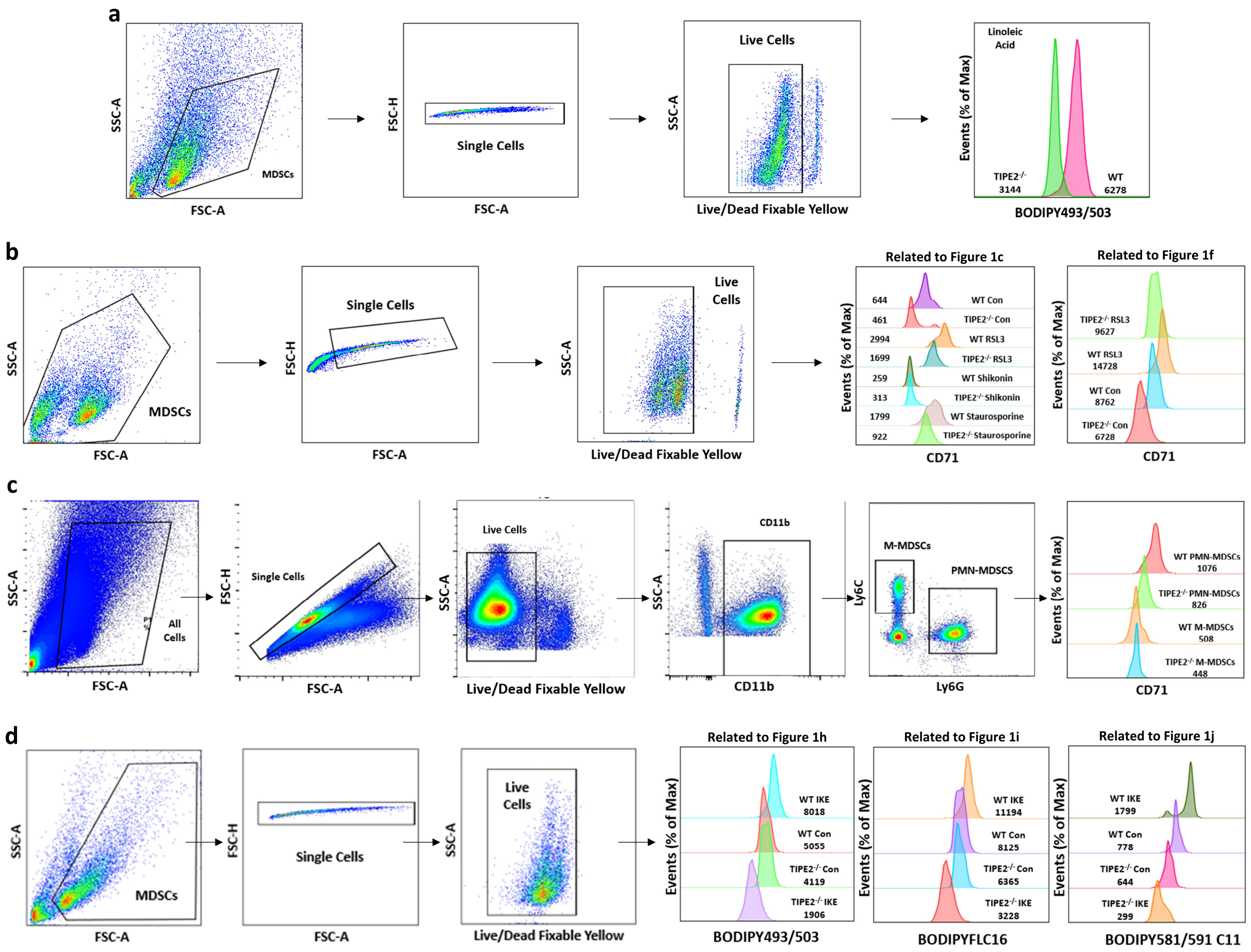

Figure S1. TIPE2 contributes to the MDSCs' activation of ferroptosis. **(a)** Flow cytometry findings demonstrating linoleic acid (polyunsaturated fatty acid, 100μM) accumulation with lipophilic fluorescent dye BODIPY493/503 in Gr-1^{+} MDSCs extracted from the bone marrow. **(b)** Flow cytometry findings demonstrating CD71 expression in Gr-1^{+} MDSCs in the presence of different cell death inducers: ferroptosis (RSL3, 20 μM), necroptosis (Shikonin, 1 μM), and apoptosis (Staurosporine, 0.25 μM). **(c)** Flow cytometry findings demonstrating CD71 expression in PMN-MDSCs and M-MDSCs extracted from the LLC TB mice. **(d)** Flow cytometry findings demonstrating the expression of indicated markers such as BODIPY493/503, BODIPYFLC16 and BODIPY581/591 C11 in Gr-1^{+} extracted from the LLC TB mice treated with IKE.

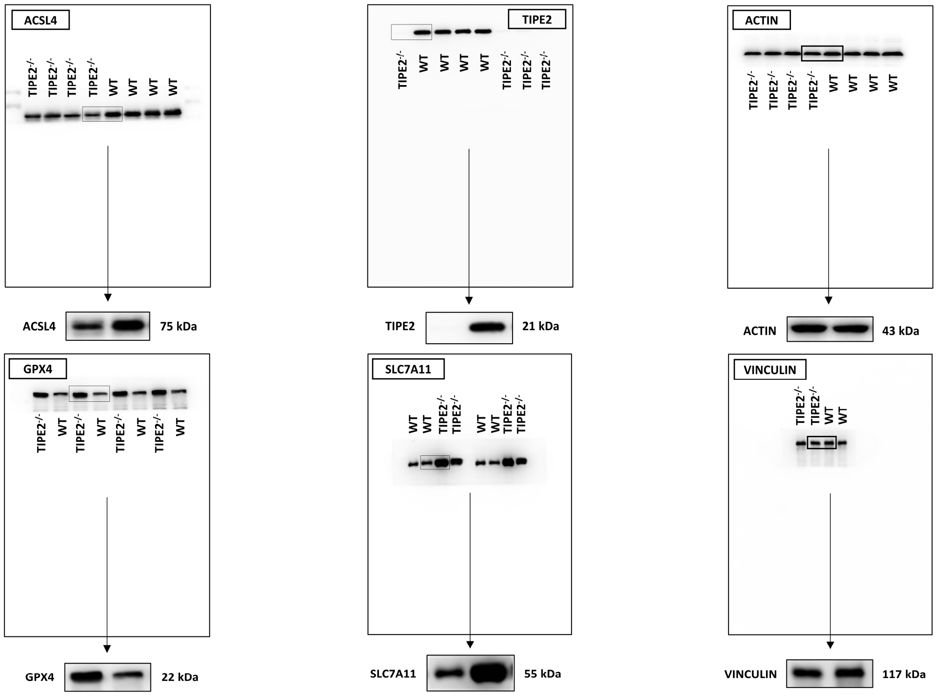

Figure S2. Immunoblot membrane findings demonstrating the TIPE2 regulation of ferroptosis-related genes of MDSCs. Western blot analysis conducted in Gr-1⁺ MDSCs extracted from the LLC TB mice treated with IKE.

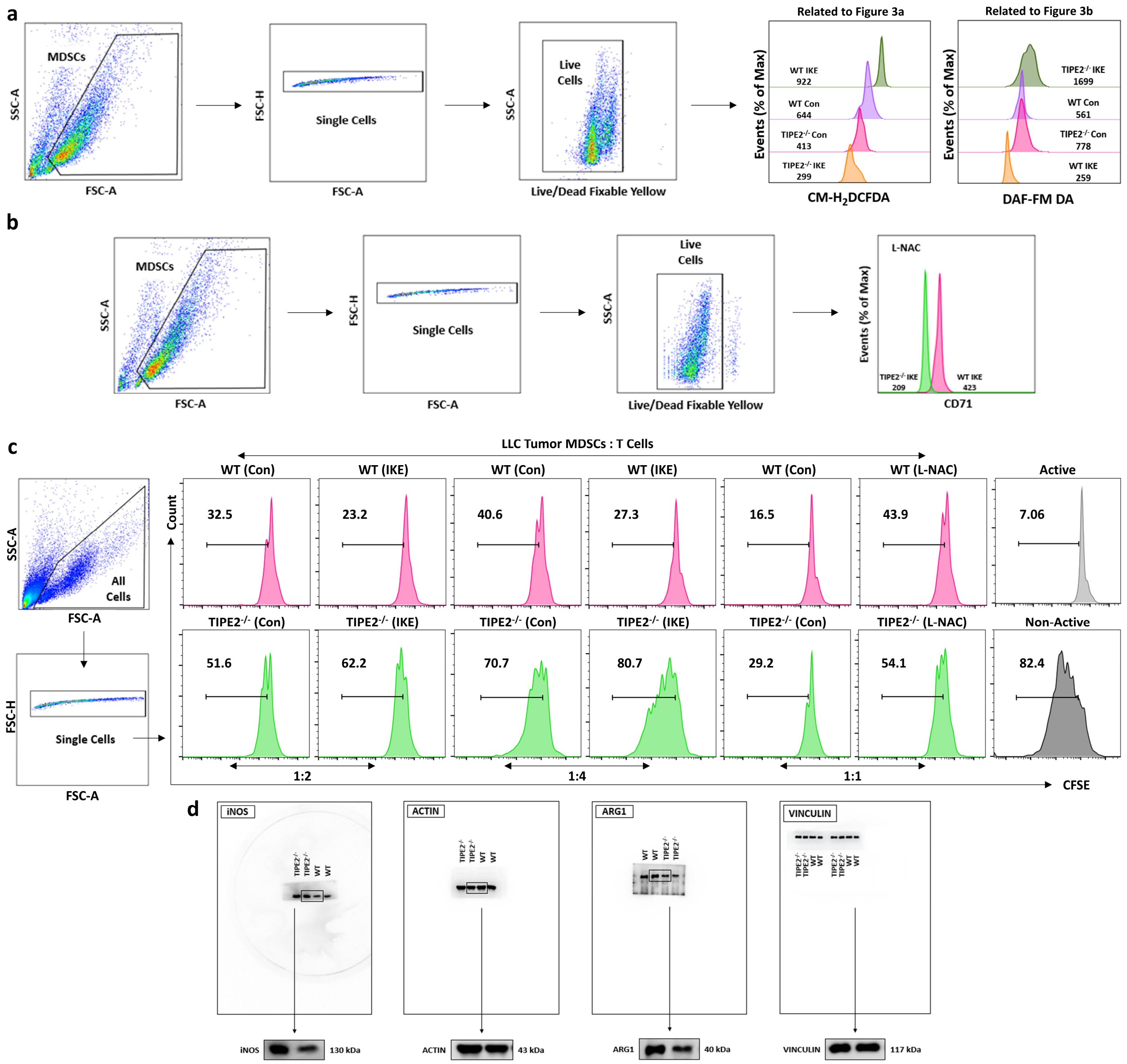

Figure S3. TIPE2-deficient MDSCs reduced the ferroptosis-induced suppressive effect. **(a)** Flow cytometry findings demonstrating the expression of indicated markers such as CM-H2DCFDA and DAF-FM DA in Gr-1+ extracted from the LLC TB mice treated with IKE. **(b)** Flow cytometry findings demonstrating CD71 expression in Gr-1+ extracted from the LLC TB mice treated with ROS inhibitor (L-NAC). **(c)** Histogram findings demonstrating the expression of T cell proliferation in Gr-1+ MDSCs extracted from the LLC TB mice treated with drugs (IKE and L-NAC) and cocultured with CFSE-labeled CD3+ T cells at the ratio of 1:1, 1:2, and 1:4. **(d)** Immunoblot membrane findings demonstrating the protein expression of ARG1 and iNOS in Gr-1+ MDSCs extracted from the LLC TB mice treated with IKE.

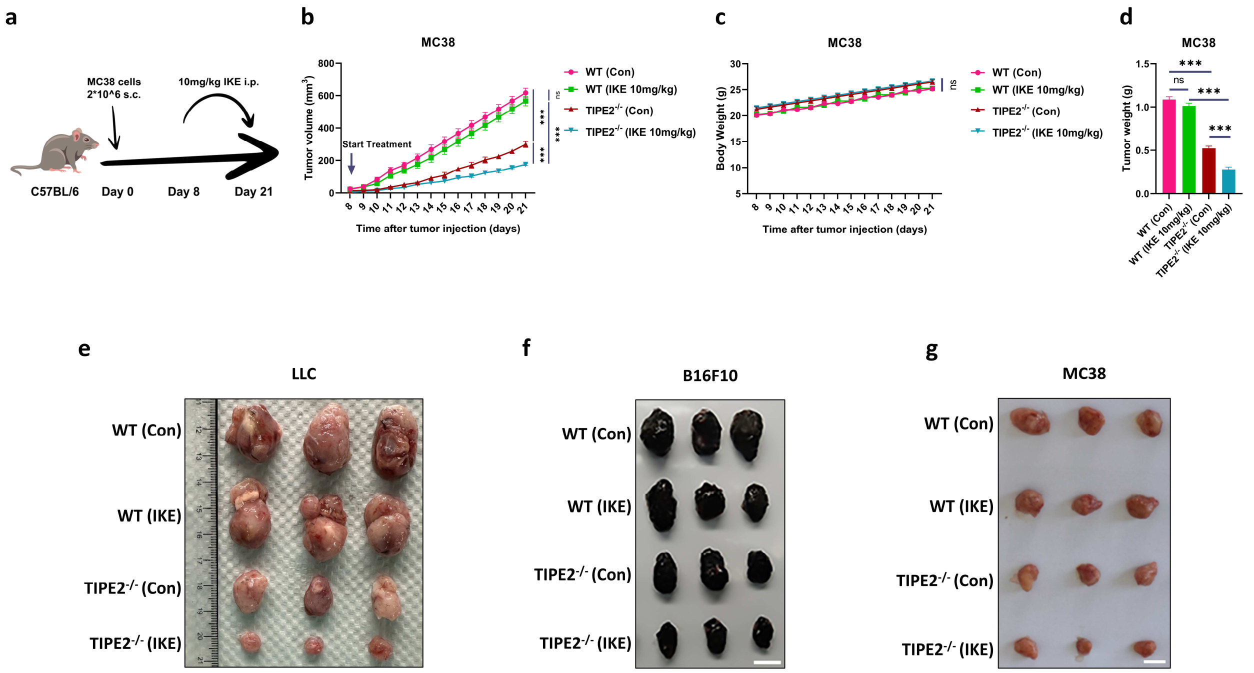

Figure S4. TIPE2-deficient MDSCs enhance ferroptosis-induced tumor growth inhibition. **(a)** A schematic representation of the experimental design C57BL/6 mice were injected s.c. with MC38 cells on day 0. Then, IKE (10mg/kg) was injected i.p. at day 8 till day 21. **(b)** The tumor volume of MC38 C57BL/6 mice (n=3 mice/group) treated for 2 weeks. **(c)** The tumor weight of MC38 C57BL/6 mice (n=3 mice/group) treated for 2 weeks. **(d)** Body weight of MC38 C57BL/6 mice (n=3 mice/group) treated for 2 weeks. **(e)** The tumor mass of LLC C57BL/6 mice (n=3 mice/group). **(f)** Tumor mass of B16F10 C57BL/6 mice (n=3 mice/group). Scale bar, 50 mm. **(g)** The tumor mass of MC38 C57BL/6 mice (n=3 mice/group). Scale bar, 50 mm.

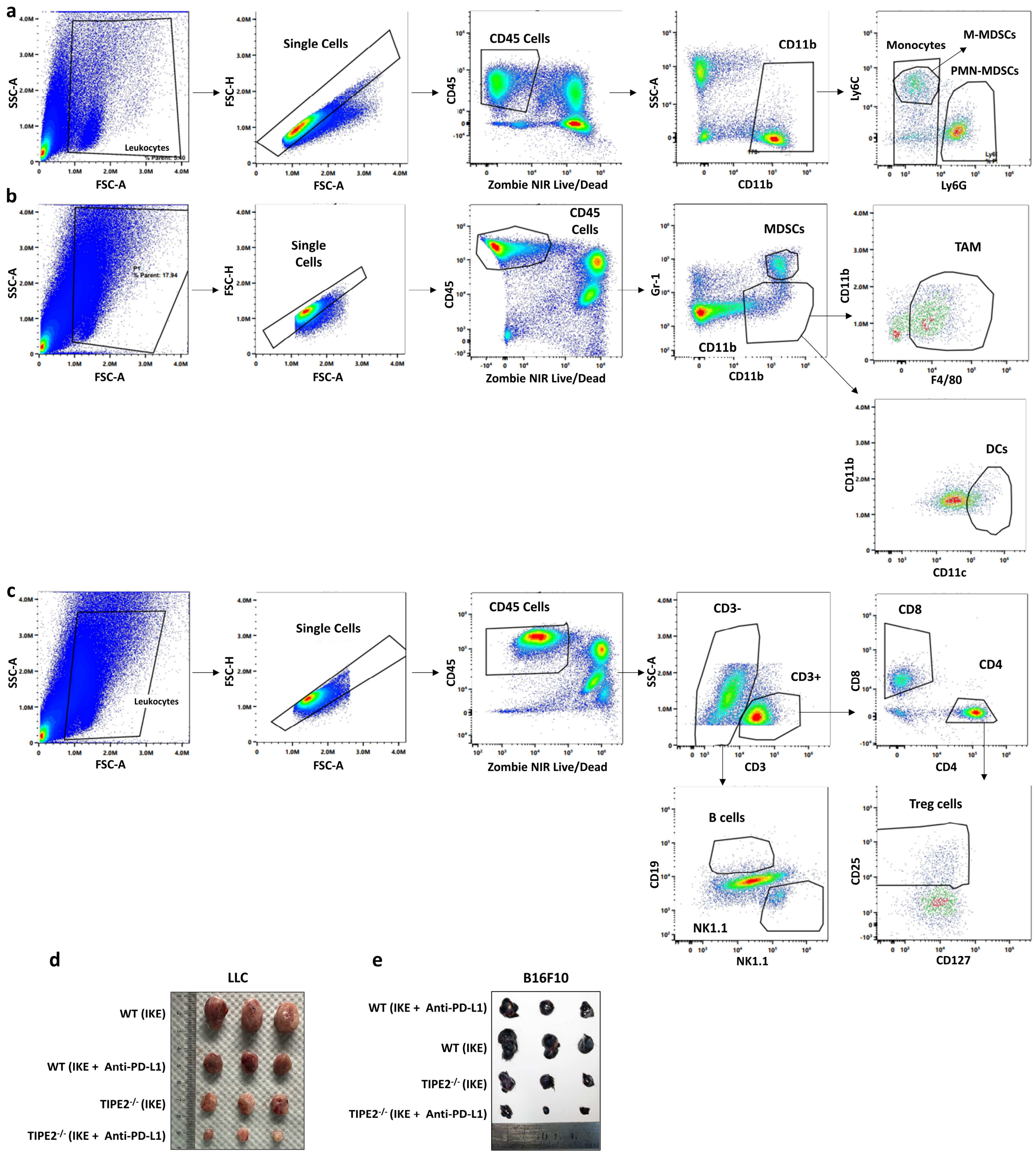

Figure S5. TIPE2-deficient MDSCs enhance ferroptosis-induced tumor growth inhibition with anti-PD-L1 cancer immunotherapy via reprogramming immune microenvironment. **(a)(b)(c)** Flow cytometry plots for leukocyte subsets in tumor-infiltrating immune cells. **(d)** The tumor mass of LLC C57BL/6 mice (n=3 mice/group). **(e)** The tumor mass of B16F10 C57BL/6 mice (n=3 mice/group).

a

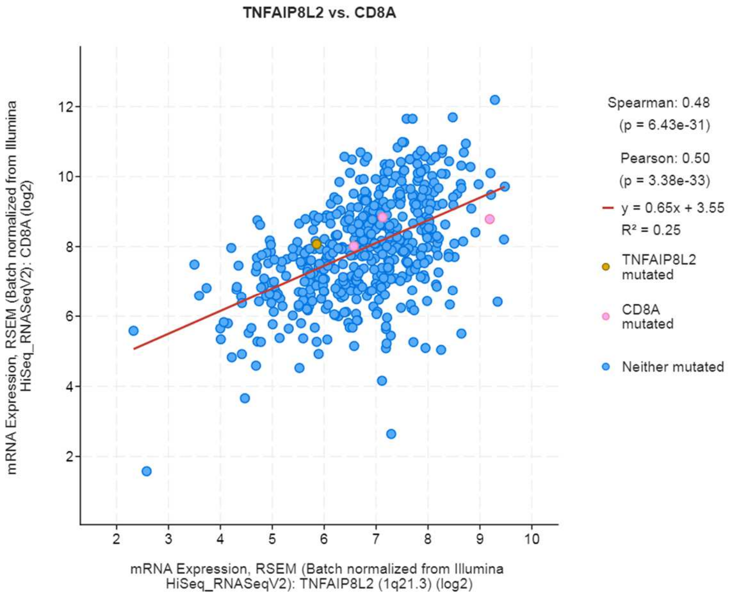

b

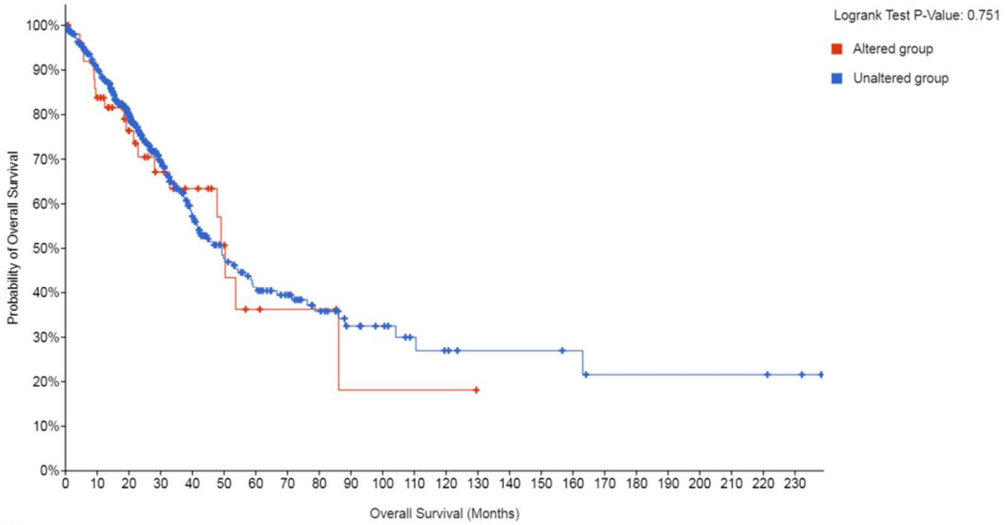

Number at risk (n)

Altered group	53	41	28	19	13	8	4	3	3	1	1	1	1	0	0	0	0	0	0	0	0	0	0
Unaltered group	452	371	247	146	95	63	49	38	27	18	15	10	8	6	6	6	5	3	3	3	3	3	2

Figure S6. There was a direct positive relationship between TIPE2 and CD8 mRNA expression in human lung adenocarcinoma in the TCGA database, but the tumor group with altered TIPE2 expression had lower overall survival probability than the unaltered tumor group. (a) We analyzed the data for TIPE2 and CD8 mRNA expression in human lung adenocarcinoma in the TCGA database using the cBioPortal website tools. (b) We compared the overall survival probability between the altered and unaltered group for TIPE2 in human lung adenocarcinoma in the TCGA database using the cBioPortal website tools.

Table S1: Primer sequences used for qPCR analyzing the gene expressions in in vitro and in vivo Gr1⁺ MDSCs.

Given names	Primer Sequences
TIPE2 Mouse Forward	CAGCGGGTGATCAAAGACCT
TIPE2 Mouse Reverse	ACCAGCTCCAGCAGAATGTC
GPX4 Mouse Forward	GCCAAAGTCCTAGGAAACGC
GPX4 Mouse Reverse	CCGGGTTGAAAGGTTCAGGA
FSP1 Mouse Forward	GAGCAACTTGGACAGCAACA
FSP1 Mouse Reverse	TTCTTCCGGGGCTCCTTATC
GCH1 Mouse Forward	AGCGCCTCACCAAACAGATT
GCH1 Mouse Reverse	TTCATTTTCTGCACGCCTCG
SLC40A1 Mouse Forward	CCCCTGCTCTGGCTGTAAAA
SLC40A1 Mouse Reverse	ATCTCGGAAAGTGCGGAAGG
DHODH Mouse Forward	ACGGACTCTATAAGCTGGGC
DHODH Mouse Reverse	TAACAGCTTGGTCCTCAGGG
SLC7A11 Mouse Forward	ATGGTCAGAAAGCCAGTTGTG
SLC7A11 Mouse Reverse	GGACAGGGCTCCAAAAAGTG
VDAC2 Mouse Forward	TCGGCAAAGCTGCCAGAGACAT
VDAC2 Mouse Reverse	GTCTCCAAGGTCCCGCTAACTT
NOX1 Mouse Forward	CTCCAGCCTATCTCATCCTGAG
NOX1 Mouse Reverse	AGTGGCAATCACTCCAGTAAGGC
PHKG2 Mouse Forward	ACCCAGGCTATGGCAAGGAAGT
PHKG2 Mouse Reverse	TGCGTAGCATCAGGATTTGGCG
SAT1 Mouse Forward	GAGGATGGCTTTGGAGAACACC
SAT1 Mouse Reverse	GATACAGCAACTTGCCAATCCATG
CHAC1 Mouse Forward	TGACCCTCCTTGAAGACCGTGA
CHAC1 Mouse Reverse	AGTGTCATAGCCACCAAGCACG
ACSL4 Mouse Forward	CCTTTGGCTCATGTGCTGGAAC
ACSL4 Mouse Reverse	GCCATAAGTGTGGGTTTCAGTAC
CARS Mouse Forward	TCATGGTGGAGGGTTTGACCTC
CARS Mouse Reverse	GGACATCTTGCAGCCTGCTATC
HO-1 Mouse Forward	CACTCTGGAGATGACACCTGAG
HO-1 Mouse Reverse	GTGTTCCTCTGTCAGCATCACC
β-Actin Mouse Forward	CGATATCGCTGCGCTGGTC
β-Actin Mouse Reverse	AGGTGTGGTGCCAGATCTTC
ARG1 Mouse Forward	GTGAAGAACCCACGGTCTGT
ARG1 Mouse Forward	AGAAAGGACACAGGTTGCCC
iNOS Mouse Forward	CACCTTGGAAGAGGAGCAAC
iNOS Mouse Forward	AAGGCCAAACACAGCATACC
